# A pre and post intervention study to reduce unnecessary urinary catheter use on general internal medicine wards of a large academic health science center

**DOI:** 10.1186/s12913-018-3421-2

**Published:** 2018-08-16

**Authors:** Krista R. Wooller, Chantal Backman, Shipa Gupta, Alison Jennings, Delvina Hasimja-Saraqini, Alan J. Forster

**Affiliations:** 1 The Ottawa Hospital, Civic Campus, 1053 Carling Avenue, Ottawa, ON K1Y 4E9 Canada; 2 Ottawa Hospital Research Institute, Clinical Epidemiology Program, 501 Smyth Box 511, Ottawa, ON K1H 8L6 Canada; 3 University of Ottawa, Faculty of Medicine 75 Laurier Avenue East, Ottawa, ON K1N 6N5 Canada; 4 School of Nursing, Faculty of Health Sciences, University of Ottawa, 75 Laurier Avenue East, Ottawa, ON K1N 6N5 Canada; 5 Ottawa Hospital Research Institute, 501 Smyth Box 511, Ottawa, ON K1H 8L6 Canada; 6 University of Ottawa, Faculty of Medicine, 75 Laurier Avenue East, Ottawa, ON K1N 6N5 Canada; 7 Clinical Epidemiology Program, Ottawa Hospital Research Institute, Civic Campus, 1053 Carling Avenue, Box 684, ASB 1st Floor, Office 1-006, Ottawa, ON K1Y 4E9 Canada; 8 The Ottawa Hospital, General Campus, 501 Smyth Rd, CPCR, Box 209, RL2136, Ottawa, ON K1H 8L6 Canada; 9 The Ottawa Hospital, Civic Campus, 1053 Carling Avenue, Executive Suites, Box 100, Ottawa, ON K1Y 4E9 Canada

**Keywords:** Urinary catheter, Patient safety, Quality improvement

## Abstract

**Background:**

Urinary catheters are a common medical intervention, yet they can also be associated with harmful adverse events such as infection, urinary tract trauma, delirium and patient discomfort. The purpose of this study was to describe the use of the SafetyLEAP program to drive improvement efforts, and specifically to reduce the use of urinary catheters on general internal medicine wards.

**Methods:**

A pre and post intervention study using the SafetyLEAP program was performed with urinary catheter prevalence as the primary outcome on two general internal medicine wards in a large academic health sciences center.

**Results:**

A total of *n* = 534 patients (*n* = 283 from ward #1; and *n* = 252 from ward #2) were included in the initial audit and feedback portion of the study and 1601 patients (*n* = 824 pre-intervention and *n* = 777 post-intervention were included in the planned quality improvement portion of the study). A total of 379 patients during the quality improvement intervention had a urinary catheter. Overall, the adherence to the SafetyLEAP program was 97.4% on both general internal medicine wards. The daily catheter point prevalence decreased from 22 to 13%. After the implementation of the program, the urinary catheter utilization ratio (defined as urinary catheter days/patient days) declined from 0.14 to 0.12. Catheter-associated urinary tract infections (CAUTI) were unchanged.

**Conclusion:**

The SafetyLEAP program can help provide a systematic approach to the detection, and reduction of safety incidents. Future studies should aim at refining and implementing this intervention broadly.

## Background

Urinary catheters are a common medical intervention; however, they can also be associated with harmful adverse events such as infection, urinary tract trauma, delirium and patient discomfort. Catheters are often placed without a justifiable indication and physicians are unaware of the presence of a non-indicated catheter 40% of the time [1]. Urinary catheters are associated with bacteriuria at a rate of approximately 5% per day and in those patients 3.6% will develop bactermia [2]. Catheter-related infections account for the majority of nosocomial infections [2]. The most effective method to reduce catheter-associated urinary tract infection (CAUTI) is to avoid unnecessary catheters and to ensure prompt removal of catheters that are no longer indicated [3].

Our institution identified urinary catheters as a patient safety issue during the implementation of the audit and feedback component of the SafetyLEAP program on our general internal medicine (GIM) wards. The SafetyLEAP program is a multimodal patient safety improvement program [4]. It is designed to engage leaders and staff to identify appropriate resources, to perform systematic measurement and to implement targeted improvement efforts. The program consists of three components that together make up the acronym LEAP: 1) **L**eadership and **E**ngagement, 2) **A**udit and feedback, and 3) **P**lanned quality improvement intervention. Audit and feedback (or prospective surveillance) is a method used to systematically identify adverse events, which may be more accurate and timely than other methods of adverse event detection [5–8]. Many organizations are still struggling to quantitatively show significant improvements in safety, therefore the use of the SafetyLEAP program to more accurately measure safety is a promising approach.

Many studies had examined the impact of evidence-based interventions on decreased catheter use and rates of CAUTI [9–13], thus our focus was on adapting these best practices using a multimodal patient safety improvement program (i.e. SafetyLEAP) as an approach to increase ward level awareness and compliance, and quantitatively demonstrate the effectiveness and sustainability of the intervention. The purpose of this study was to describe the use of the SafetyLEAP program to drive improvement efforts, and specifically to reduce the prevalence of urinary catheters at a large academic health center.

## Methods

### Design and setting

**Fig. 1:**
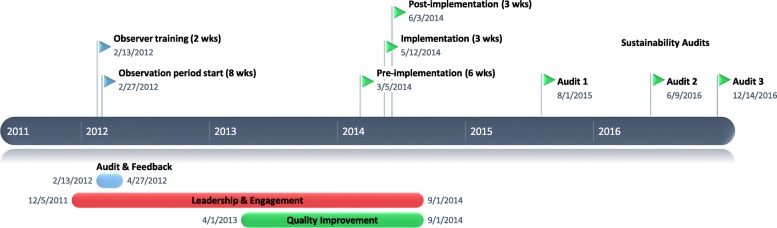
Timeline of SafetyLEAP intervention

A pre and post intervention study using the SafetyLEAP program was performed to reduce urinary catheter use on two GIM wards in a large academic health sciences center. The study was conducted at a tertiary care teaching hospital consisting of approximately 140 general medicine beds, which include 14 intensive observation beds. The study population included all the inpatients on two GIM wards at the time of the study. Study procedures were reviewed and approved by the local research ethics board. The first 2 components of the SafetyLEAP program were carried out 2012–2013. The third component was planned in 2013 and carried out in over 4 months in 2014 (Fig. 1).

### Components of the SafetyLEAP program

The SafetyLEAP Program consisted of the following components:

1. **Leadership and** **Engagement:** This component consisted of specific methods to engage leaders, and healthcare providers in the program planning and execution. The specific activities as part of this component included: 1) obtaining *leadership support, 2)* identifying a *local champion* and other *key project members*, 3) developing a *communication plan*, and confirming *appropriate resources*. We ensured that we had a leader within our institution with enough authority to provide accountability to senior management for the wards’ participation in the project as well as a dedicated ‘project lead’, and a project team. The project team was directly involved with the development and implementation of the communication plan, and the introduction of the program to key stakeholders and staff. This included presence on the ward to provide information about the study and answer questions, providing key messages through ward presentations, emails, and posters to staff and residents to inform them about the study. Specific resources were also secured for the project including a clinical observer and clinical reviewer for the audit and feedback.
2. **Audit and feedback:** Prospective surveillance of the GIM population in our institution has been described in previous research [5]. In brief, a healthcare provider was recruited to be an observer to monitor care and look for specific triggers such as pharmacy orders, system events, clinical phenomenon, laboratory results that could be indicative of an incident. The project team defined the parameters and the *triggers* to include for the audit and feedback. A *two-week training* period was provided during which time information about adverse events, clinical observations, and mock cases were presented and discussed. Staff had opportunities to help identify potential patient or staff safety issues during the audit. Following the orientation, the observer conducted daily audits for an 8-week period *(case findings).* Baseline information was captured for all patients on the ward. To detect safety events, each patient was monitored using daily chart reviews, interactions with clinicians, and direct observations. The observer also identified and documented any events occurring when off-duty by using chart review or voluntary reports from other staff. On a weekly basis, a *clinical team* (i.e. physician and nurse) reviewed each case (including a brief description, the actions taken, and the outcomes) to assess if the event was an adverse event, potential adverse event and whether the event was preventable or not *(case reviews).* Following the reviews, a designated core review team classified all the events for type and severity based on the WHO event classification *(case classification). Results* of the audit were shared with the healthcare team for discussion and identification of priority problems.
3. **Planned quality improvement intervention:** Following the review of the audit and feedback results that highlighted catheters as an area of concern, a *local quality improvement team* was selected to tackle this issue. The team was requested to lead the CAUTI improvement effort using the Model for Improvement (Plan-Do-Study-Act). The team was asked to document the plan using a project charter, implement the plan, monitor using run or control charts, summarize the results and decide to make further improvements or discontinue the plan. Urinary catheter point prevalence, catheter utilization ratio and unnecessary catheter utilization ratios were the primary outcomes examined. During the quality improvement intervention, daily audits of the catheter status were completed by a research assistant. Catheters were determined to have a justifiable indication if they were being used for acute kidney injury, close monitoring of ins and outs, urinary retention or bladder outlet obstruction, gross hematuria requiring bladder irrigation, prevention of wound contamination, palliative care, unstable spine or pelvic injury requiring immobilization or if patient had a chronic indwelling catheter on admission. If no identifiable indication was present they were determined to be unnecessary. Pre-implementation data was collected for 6 weeks. There was 4 weeks of educations about the intervention during which no data was collected. The intervention was implemented over the first 3 weeks of the post-intervention period and modified with PDSA cycles. The post-intervention period continued for an additional 3 weeks during which catheter prevalence was monitored. Daily measurement of catheter prevalence was monitored with a run chart during the quality improvement intervention and calculated based on the total number of catheters present divided by the total number of patients on the ward. Calculation of catheter utilization ratios were based on patients present on the ward during the pre-implementation data collection and post-implementation data collection period respectively. Patients who were admitted continuously through the pre- and post-implementation periods were excluded from the analysis of catheter utilization rates.

### Definitions

The Infectious Diseases Society of America guidelines were used to define CAUTI and CA-ASB [14, 15]. According to these guidelines, signs or symptoms of CAUTI include new onset or worsening fever, rigors, altered mental status, malaise, or lethargy without another identifiable cause; flank pain; costovertebral angle tenderness; acute hematuria; pelvic discomfort; and dysuria, urgency, frequency in a patient whose catheter has been removed. Management of CAUTI was defined as appropriate if the patient was prescribed an antimicrobial and as inappropriate if no antimicrobial was prescribed. According to the IDSA guidelines, a positive urine culture corresponded to any organism growth of at least 10^5^ CFU/mL, or any organism growth of at least 10^3^ CFU/mL in association with one of the following: 1) positive dipstick for leukocyte esterase and/or nitrates, 2) pyuria (urine specimen with ≥10 WBC/mm^3^ of unspun urine or > 5 WBC/ high power field on spun urine) or 3) positive gram stain of unspun urine.

CA-ASB was defined by positive urine culture, consistent with the definition of CAUTI, without signs and symptoms. Management of CA-ASB was defined as appropriate if no antimicrobial was prescribed and as inappropriate if an antimicrobial was prescribed.

### Statistical analysis

To evaluate the SafetyLEAP program, we conducted an independent audit of the program’s metrics for their completeness. The auditor was a Masters level Research Assistant from our institution with experience in auditing safety practices. The five criteria from the *Leadership and engagement* component included: (1) an executive leader who has agreed to the project, (2) a project lead, (3) a project team, (4) the implementation of a communication plan, and (5) the identification of adequate resources.

The seven criteria for the *Audit and feedback* component included: (1) completion of training sessions for the clinical observer and clinical reviewer, (2) involvement of the project team in decisions concerning which triggers to include, (3) completion of the audit period, (4) review of all cases, (5) presence of a minimum of one physician and one other health professional at 75% of the review meetings, (6) classification of all cases and preparation of the report, and (7) review of the final results by the project team.

The seven criteria for the *Quality improvement* component included: (1) identification and documentation of a priority problem, (2) identification of a quality improvement team (minimum of 2 people), (3) documentation of the plan using a tool such as a project charter, (4) implementation of the plan, (5) monitoring the plan using run or control charts, (6) communication of the results, and (7) deciding whether to make further improvements or to discontinue the plan. For each component of the program, and for the program overall, a proportion of completeness was provided.

Catheter utilization ratios for each period were calculated by dividing the total number of catheter-days by the total number of patient-days. The proportion of unnecessary catheter days was calculated by dividing the total number of unnecessary catheter days by the total number of catheter-days. CAUTI and CA-ASB rates were calculated by measuring the incidence of each diagnosis through the study period divided by the number of catheter days for each study period and multiplying by 1000. Rate ratios and 95% confidence intervals and *p* values based on Mid-P exact test were determined for each rate. Mean LOS and age were calculated using log transformed and squared- transformed data respectively and t-tests used to calculate the p value for differences between the pre and post group. Pearson Chi squared test was used to calculate p values for differences between proportion of female patients, hospital site, ward of catheter insertion, catheter utilization ratio and unnecessary catheter utilization percentage between the pre and post groups. All statistical analysis was performed using Stata IC v.14 statistical software.

## Results

The study took place between February 2012 and June 2014. Overall, the SafetyLEAP program was implemented successfully (97.4%) on both (*n* = 2) GIM wards. The results of the program implementation can be found in Table 1, and further described below.

**Table 1:** Adherence with program

Activity	POINTS	
	Yes = 1, Partial = 0.5, No = 0	
	GIM ward #1	GIM ward #2
ENGAGEMENT	100.00%	100.00%
Leadership support	1	1
Local champion	1	1
Other key project members	1	1
Communication plan	1	1
Resources provided	1	1
AUDIT AND FEEDBACK	92.9%	92.9%
Audit and feedback training	1	1
Audit triggers confirmed	1	1
Case findings	1	1
Case reviews	1	1
Composition of multidisciplinary review team	0.5	0.5
Results summarized (including case classification)	1	1
Results reviewed	1	1
QUALITY IMPROVEMENT	100.00%	100.00%
Plan - Priority selected	1	1
Plan - Local QI team identified	1	1
Plan - Plan to test the change documented	1	1
Do - Plan implemented	1	1
Study - Results monitored	1	1
Act - Results summarized	1	1
Act - Decision taken on next steps	1	1
FULL PROGRAM	97.4%	97.4%

1) Leadership andEngagement: This component was implemented at 100% on both (n = 2) GIM wards. During the study, a senior leader, ward-based dedicated project leads (KW and DH), and project team members were identified. A local team was identified to facilitate the development and the implementation of the project-related communication plan for stakeholders/staff. Specific resources (including a clinical observer and a clinical reviewer) were committed to the project.

2) Audit and feedback.

A total of 534 unique patients (*n* = 283; ward #1, *n* = 252; ward #2) were included in the study during the audit and feedback period. The audit and feedback took place between February and April 2012 by a trained observer. The average patient age was 69.1 years and 51.9% of patients were female. The patient characteristics are described in Table 2.

**Table 2:** Patient characteristics during audit and feedback

Variable	Ward # 1	Ward # 2	All Sites
Patient encounters, N	298	263	561
Unique Patients Observed, N	283	252	534^a^
Female, N (%)	160 (53.7%)	131 (49.8%)	291 (51.9%)
Patient Age, mean (median)	71.5 (76)	66.5 (69)	69.1 (73)
Days Observed per Encounter, average (median)	9.6 (7)	8.6 (6)	9.1 (6)
Admitting diagnoses (10 most common)			
Pneumonia	27 (9.1%)	34 (12.9%)	61 (10.9%)
Congestive Heart Failure	13 (4.4%)	16 (6.1%)	29 (5.2%)
COPD exacerbation	19 (6.4%)	4 (1.5%)	23 (4.1%)
Sepsis	15 (5.0%)	6 (2.3%)	21 (3.7%)
Cellulitis	11 (3.7%)	9 (3.4%)	20 (3.6%)
Failure to cope	8 (2.7%)	11 (4.2%)	19 (3.4%)
Acute Renal Failure	7 (2.3%)	9 (3.4%)	16 (2.9%)
Delirium	6 (2.0%)	9 (3.4%)	15 (2.7%)
GI bleed (Upper)	10 (3.4%)	5 (1.9%)	15 (2.7%)
Weakness	9 (3.0%)	4 (1.5%)	13 (2.3%)
Comorbidities (10 most common)			
Hypertension	154 (51.7%)	109 (41.4%)	263 (46.9%)
Diabetes Mellitus Type 2	76 (25.5%)	68 (25.9%)	144 (25.7%)
Atrial Fibrillation	68 (22.8%)	40 (15.2%)	108 (19.3%)
Congestive Heart Failure	55 (18.5%)	34 (12.9%)	89 (15.9%)
Chronic Obstructive Pulmonary Disease	53 (17.8%)	36 (13.7%)	89 (15.9%)
Chronic Kidney Disease	44 (14.8%)	33 (12.5%)	77 (13.7%)
Dyslipidemia	33 (11.1%)	37 (14.1%)	70 (12.5%)
Gastroesophageal Reflux Disease	30 (10.1%)	33 (12.5%)	63 (11.2%)
Hypothyroidism	32 (10.7%)	29 (11.0%)	61 (10.9%)
Dementia	29 (9.7%)	28 (10.6%)	57 (10.2%)

^a^Note: One patient was observed at both campuses. 12 encounters do not have comorbidities entered

The audit and feedback identified 299 adverse events on both the GIM wards. The most common class of events was related to clinical processes/procedures followed by healthcare associated infections. Overall, 6.6% of the events (20/299) were directly related to urinary catheters and included: trauma, infection, bleeding secondary to catheter use, failure to promptly remove unnecessary catheters, and no clear indication for catheter use (Table 3). There was one patient death associated with a complication from a urinary catheter.

**Table 3:** Catheter associated adverse events during audit and feedback – classification

WHO Level 1 Classification	Total Events*	Urinary catheter events*	Preventable AE*	Non-Preventable AE*	Potential AE*
Clinical process/procedure	148 (49.5%)	14 (70%)	8 (61.5%)	3 (75%)	3 (100%)
Healthcare associated infection	28 (9.4%)	5 (25%)	5 (38.5%)	0 (0%)	0 (0%)
Medication/IV fluid/biological (includes vaccine)	79 (26.4%)	1 (5%)	0 (0%)	1 (25%)	0 (0%)
Other	44 (14.7%)	0	–	–	–
Total	299 (100%)	20 (100%)	13 (100%)	4 (100%)	3 (100%)

^*^Percentages are relative to column totals

All of the cases were reviewed during weekly meetings with a physician and a clinical observer, but without another healthcare professional present – as specified in the SafetyLEAP protocol. After the audit and feedback period was completed, general care including processes for catheter care were identified as a key priority and led to focused work to identify reasons for catheterization on the wards as well as an improvement project to decrease unnecessary urinary catheterization. Overall, 92.9% of the audit and feedback tasks were completed on both GIM wards – the only omission was the failure to include a professional other than a physician in the weekly review session.

3) Planned quality improvement intervention.

**Fig. 2:**
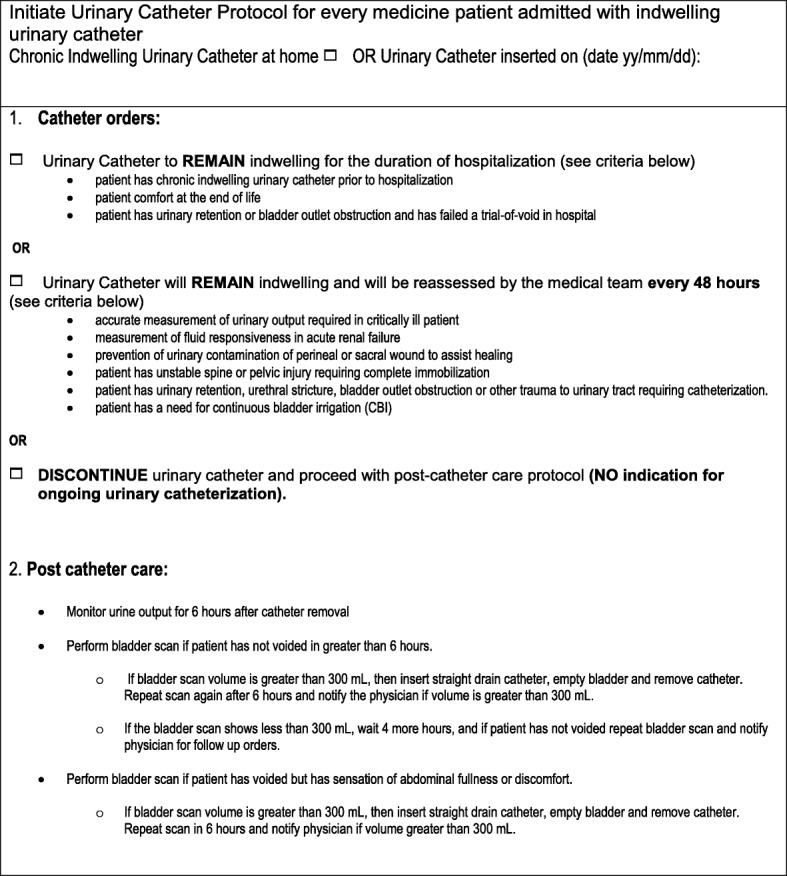
Urinary catheter protocol

Urinary catheters were targeted for improvement based on their contribution to overall adverse events on the GIM wards. Based on a literature review of accepted criteria for catheterization, a standardized order sheet was prepared to indicate appropriate use of indwelling urinary catheters. As per the indwelling catheter order sheet (Fig. 2), the catheter was to remain in place if (1) the patient required it for end of life comfort, (2) the patient had a chronic indwelling catheter prior to hospitalization, or (3) the patient had urinary retention or bladder outlet obstruction and had failed a trial-of-void in hospital. The catheter was left in place and reassessed in 48 h if the patient required accurate measurement of urinary output (in critically ill patients), measurement of fluid responsiveness in acute renal failure, prevention of urinary contamination of perineal or sacral wound to assist healing, patient with unstable spine or pelvic injury requiring immobilization, patient with urinary retention, urethral stricture, bladder outlet obstruction or other trauma to urinary tract requiring catheterization, or patient requiring continuous bladder irrigation. As per the indwelling catheter order sheet, if none of the indications were met, then the urinary catheter was discontinued and post-catheter care was initiated. After the catheter removal, urine output was observed for the first 6 h. If there was no urine output in the first 6 h, then a bladder scan was done, and if the bladder scan showed bladder volume greater than 300 ml then in-and-out catheterization was done, with 6-h post repeat bladder scan. The inclusion of a formal protocol for in-and out catheterization was part of a catheter order set and a new initiative in our institution.

The catheter order sheet was implemented after training for nurses, residents and physicians was conducted. Education consisted of posters in the resident and nursing rooms, nursing education sessions with the ward nurse educators, and a presentation to the residents rotating through the wards as well as the internal medicine residents during their protected academic time. Presentations were repeated when new residents rotated through the teaching wards and included the findings from the audit and feedback. Nursing staff or ward clerks inserted an order sheet in the charts of all medicine in-patients with indwelling urinary catheters. Clinical care leaders were prompted to ask about urinary catheters in place during daily bullet rounds and to remind the nurses and physicians to use the protocol. Urinary catheter audits were continued during the implementation period (3 weeks) and post-implementation period (3 weeks).

**Fig. 3:**
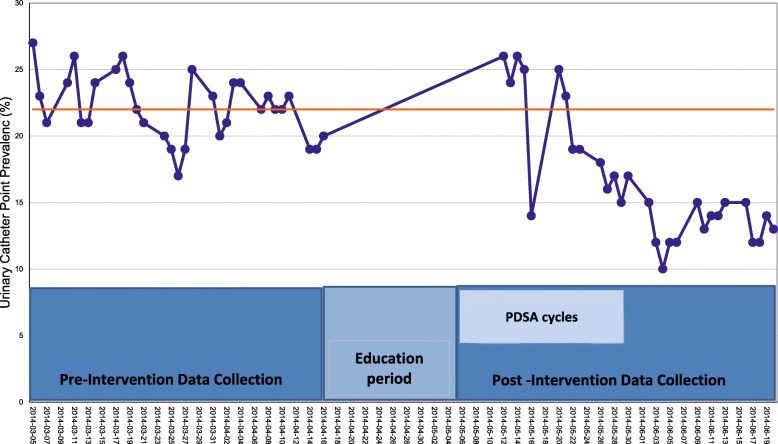
Run chart of urinary catheter point prevalence during planned quality improvement

The characteristics of patients audited in the pre-intervention period and post-intervention period are outlined in Table 4 and the groups had similar age, gender and length of stay. Urinary catheter prevalence prior to the implementation was 22.4% over a 6-week period as measured by daily audits (Fig. 3). Most catheters were placed in the emergency room (67%).

**Table 4:** Patient characteristics during quality improvement intervention

	Pre-intervention (N = 824)	Post-interventions (N = 777)	*P*
Female, N(%)	415 (50.4)	417 (53.7)	*p* = 0.186
Ward 1, N(%)	388 (47.1)	371 (47.8)	*p* = 0.791
Age, mean (median, IQR)	73.0 (74, 61–85)	72.6 (75,58–85)	*p* = 0.618
Length of stay, mean (median)	7.0 days (7)	6.6 days (6)	*p* = 0.169

After implementation of the order set, the daily prevalence rate dropped to 13.0% over 3-week post-implementation (Fig. 3). Sustainability audits conducted on the GIM wards at both the one-year and two-year mark post-implementation, showed the prevalence was sustained at 15.0%. Post-intervention, 59% of catheters were inserted in the emergency room.

The urinary catheter utilization ratio and proportion of unnecessary urinary catheter days were calculated for patients in the pre- and post-intervention groups and are shown in Table 5. The catheter utilization ratio decreased from 0.144 to 0.116. Unnecessary catheters decreased from 35 to 27%. During the last phase of the quality improvement intervention (post-implementation) the catheter utilization ratio further fell to 0.09 and the proportion of unnecessary catheter days fell to 16%.

**Table 5:** Catheter Utilization Ratio, Unnecessary Catheter Utilization Ratio, CAUTI and CA-ASB before and after catheter protocol introduction

	Pre-intervention (N = 824)	Post-intervention (N = 777)		
Catheter days	952	716		
Patient days	6617	6167		
Catheter Utilization Ratio	0.144	0.116	*p* < 0.0001	
Unnecessary Catheter Utilization %	35.0	26.7	*p* < 0.0003	
CAUTI rate – incident CAUTI per 1000 catheter-days (N)	21 (20)	24 (17)	Rate ratio 1.13 (95% CI 0.56–2.3)	*p* = 0.710
CA-ASB rate – incident CA-ASB per 1000 catheter-days (N)	20 (19)	9.8 (7)	Rate ratio 0.49 (95% CI 0.17–1.2)	*p* = 0.101
CAUTI rate with appropriate antibiotics (N)	19 (18)	22.3(16)	Rate ratio 1.18 (95% CI 0.56–2.4)	*p* = 0.628
CA-ASB rate treated with antibiotics (N)	14.7 (14)	2.8 (2)	Rate ratio 0.19 (95% CI 0.02–0.83)	*p* = 0.012

CA-ASB rate decreased after implementation although the result did not meet statistical significance (Table 5). Antibiotic prescription for asymptomatic bacteria significantly decreased however, CAUTI rates did not change through the intervention.

## Discussion

The SafetyLEAP program aims to drive not only the systematic detection of safety events, but also to provide the tools required to focus on improvement efforts to reduce or minimize safety incidents. In this study, implementation of the SafetyLEAP program resulted in the identification of inappropriate urinary catheter use as a key issue on the GIM wards. The planned quality improvement facet (the P in LEAP) of the SafetyLEAP program was then used to develop and implement several evidence-based interventions to reduce the use of urinary catheters on these wards. The effect of this quality improvement project was sustained over two years. In reviewing the implementation of the SafetyLEAP program on these wards, it seems likely the excellent uptake was a function of physician leadership buy-in as well as evidence-based standard post-catheter care orders, both of which were regarded favourably by physicians and nurses alike.

Despite the reduction in inappropriate catheter use on the GIM wards, catheter-associated urinary tract infections were not statistically changed during our intervention. In reviewing the run chart monitoring the intervention, real changes in catheter prevalence did not appear until the last three weeks of the measurement period. The overall number of CAUTI detected during our study was small and it is possible that with further surveillance and continued decreased catheter prevalence we may have seen a decrease in infections. In addition, as we observed in the audit and feedback component of our intervention, urinary catheters were associated with other complications such as urethral trauma. While we did not track these events during the planned quality improvement phase, these would also likely be reduced with decreased catheter utilization. Interestingly, it appeared that antibiotic prescribing for CA-ASB decreased with the intervention. Efforts aimed at reducing unnecessary urinary catheters may be an important way to improve antibiotic stewardship.

Audit and feedback (or prospective surveillance) may be a more reliable and timely method to identify adverse events in hospital. One of the benefits is the ability to provide credible data to support real time changes in practice [16]. Other investigators have suggested that although prospective surveillance is useful for identifying and characterizing preventable adverse events, the findings are too heterogeneous to inform specific improvement interventions [17]. No adverse event surveillance system will lead to real change unless it is coupled with a follow-up improvement intervention. Linking of ward specific adverse event data with leadership engagement and improvement plans can be a powerful motivator for change and likely a key element in the success of our project. Furthermore, other key elements to our success included: relevant and timely local data, which helped make a case for change, leadership support, and the engagement of physicians and nursing staff. Importantly, the team involved in this planned quality improvement effort volunteered to work on this project as opposed to being assigned to this task. The team consisted of nurse educators, attending physicians, and resident physicians. There was also financial support, which facilitated the hiring of an assistant to perform audits during the planned intervention. The resident physicians were able to present the results of the work in scholarly venues (i.e. resident research day and the hospital patient safety conference), which greatly increased interest in the project among resident physicians.

There were several challenges that we encountered. Firstly, we found that most of the inappropriate catheters were being placed in the emergency room without a physician order. Future work will look at targeting this aspect to further reduce the burden of inappropriate catheterization. Secondly, the reassessment of initially indicated urinary catheters every 48 h did not seem to be effective. Other investigators have found that nurse led reminders can be effective [18] and future work will incorporate this into our protocol. Our goal is to expand our study across our institution and continue to test and trial solutions to this problem as we expand.

Limitations to the current study include the observational nature of the study. There may have been other unaccounted for factors that contributed to the decrease in urinary catheter prevalence and decrease in antibiotic prescribing for CA-ASB. Furthermore, the mere presence of auditors may have improved the results and caused an overestimate of the effectiveness of the intervention.

## Conclusion

The SafetyLEAP program can help hospital wards target improvement projects to improve safety. We used it to identify leaders’ engaged in safety, perform a safety audit to determine the most important threat to safety, and respond to a challenge (specifically urinary catheter associated harm). This disciplined approach is reproducible and can itself be audited. This provides leaders a method to systematically address patient safety issues in their institution, while at the same time create continuous learning on the capacity of their wards to improve. Future studies should further explore whether performance on the SafetyLEAP audit tool is associated with successful reduction of safety threat.

## Abbreviations

CA-ASB: Catheter-associated asymptomatic bacteriuria
CAUTI: Catheter-associated urinary tract infection
GIM: General internal medicine
LEAP: Leadership and Engagement, Audit and feedback, Planned quality improvement
PDSA: Plan, Do, Study, Act
